# Dynamic shifts in pathogen ecology of catheter-related bloodstream infections: temporal trends and ward-specific risk landscapes

**DOI:** 10.3389/fmed.2025.1665350

**Published:** 2026-01-23

**Authors:** Shanfeng Li, Jia Zuo, Yu Xie, Weifen Liu, Hanjie Yi

**Affiliations:** 1 Department of Nosocomial Infection Management, The Second Affiliated Hospital of Nanchang University, Nanchang, China; 2 Information Office, The Second Affiliated Hospital of Nanchang University, Nanchang, China; 3 Department of Oncology, The Second Affiliated Hospital of Nanchang University, Nanchang, China

**Keywords:** APACHE II, catheter-related bloodstream infection, mortality, multidrug-resistant organisms, *Staphylococcus aureus*

## Abstract

Catheter-related bloodstream infections (CRBSIs) represent a severe clinical complication with high mortality and pose a significant public health challenge due to increasing multidrug-resistant organisms (MDROs). This study aimed to systematically analyze the epidemiology, pathogen distribution, and independent predictors of 28-day mortality in CRBSI to support early risk warning and precise intervention. A retrospective cohort study included 172 patients with confirmed CRBSI, defined per CDC criteria. Data were extracted from electronic health records. Blood cultures used the BACT/ALERT^®^ 3D system; pathogen identification and antimicrobial susceptibility testing utilized the VITEK^®^2 COMPACT platform. Statistical analysis with SPSS 26.0 employed univariate and multivariate logistic regression to identify mortality risk factors, including evaluating a nomogram model for predictive performance. The 28-day mortality rate was 19.77%. Epidemiological surveillance revealed a significant year-by-year decline in CRBSI incidence from 2021 to 2024 (*p* < 0.01). High-risk wards included Nephrology (34.88%), ICU (22.67%), and Gastroenterology (12.21%), accounting for 69.76% of cases. Gram-positive microorganisms predominated (51.74%, 89/172), with *Staphylococcus aureus* as the leading pathogen (41.28%, 71/172); *Escherichia coli* and *Klebsiella pneumoniae* showed significant increasing trends (*p* < 0.05). Independent predictors of 28-day mortality were APACHE II score (OR = 1.771, 95% CI: 1.328–2.360) and cardiovascular disease (CVD) (OR = 19.426, 95% CI: 1.248–52.270); among microbiological variables/MDROs, only carbapenem-resistant *Acinetobacter baumannii* (CR-AB) infection (OR = 3.549) and carbapenem-resistant *K. pneumoniae* (CR-KP) infection (OR = 5.301) remained independently associated with mortality, while Gram-positive microorganism infection was protective (OR = 0.081). The nomogram demonstrated excellent predictive performance (C-index = 0.979), identifying APACHE II score as the most influential predictor; ROC analysis confirmed disease severity as the core mortality determinant. Findings confirm APACHE II score and CVD are strong mortality predictors, while Gram-positive infections correlate with favorable outcomes. Strengthening infection control effectively reduced incidence, highlighting the need for enhanced surveillance in high-risk departments and continuous monitoring of pathogen distribution and antimicrobial resistance, with particular emphasis on carbapenem-resistant Gram-negative organisms. These results support risk stratification and individualized treatment, though multicenter validation remains necessary.

## Graphical Abstract

**Figure:**
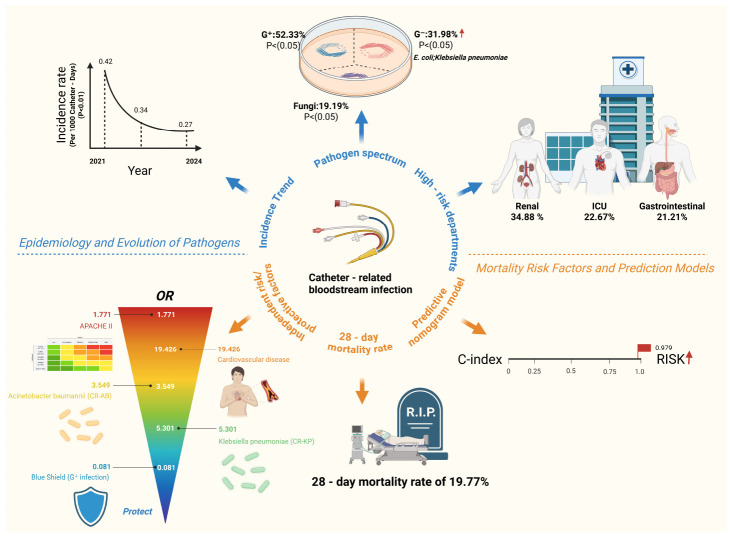
Overview of the epidemiological evolution and 28-day mortality risk in catheter-related bloodstream infections (CRBSIs). Created with BioRender.com.

## Highlights

- This study is the first to report the epidemiological characteristics of CRBSIs and their association with MDRO infections in the Nanchang region.
- The study identifies the APACHE II score as a strong predictor of 28-day mortality in patients with CRBSIs.
- CVD is found to significantly increase the risk of mortality in patients with CRBSIs.
- Infections caused by Gram-positive bacteria are associated with a lower mortality rate.
- This study provides critical evidence for the clinical management, prevention, and prognostic evaluation of CRBSIs.

## Introduction

Catheter-related bloodstream infections (CRBSIs) are a common and serious form of hospital-acquired infection,

particularly among critically ill patients in intensive care units (ICUs) and nephrology wards, and have become a major focus of infection prevention worldwide (1–3). CRBSIs usually result from microbial colonization of central venous catheters during insertion or maintenance, leading to bloodstream invasion, systemic inflammatory response, sepsis and even death (4, 5). Their clinical presentation is often insidious, and early diagnosis is challenging, increasing the risk of delayed treatment and disease progression (6). Although the implementation of aseptic techniques and catheter-care bundles has contributed to an overall decline in CRBSI incidence (7), these infections still occur in high-risk units, prolong hospital stay, increase healthcare costs and are associated with substantial short-term mortality, especially in patients with significant comorbidities (8–11).

In recent years, the rising burden of multidrug-resistant organisms (MDROs) in CRBSIs has attracted increasing attention (12). Carbapenem-resistant *Acinetobacter baumannii* (CR-AB) and carbapenem-resistant *Klebsiella pneumoniae* (CR-KP) spread rapidly in hospital settings, severely constraining therapeutic options and markedly worsening outcomes (13–16). MDROs frequently exhibit enhanced virulence and a pronounced capacity for biofilm formation; biofilms on catheter surfaces substantially increase bacterial tolerance to antibiotics and host immune clearance, rendering infections difficult to eradicate and prone to relapse, and predisposing to uncontrolled systemic infection and multi-organ failure (17–19). These features are particularly relevant in CRBSIs, in which catheter-associated biofilm serves as a reservoir for MDRO colonization and recurrent bloodstream seeding. By contrast, some studies suggest that CRBSIs caused by Gram-positive organisms may be associated with relatively more favorable outcomes at the population level (20). However, MRSA represents a notable exception: owing to its multidrug resistance and high virulence, MRSA bacteremia is consistently linked to increased mortality and cannot be considered prognostically benign (21, 22). In parallel, several epidemiological studies have reported a year-on-year increase in infections due to resistant Gram-negative bacteria, indicating a dynamic shift in the pathogen spectrum (23). This trend also applies to CRBSIs, where carbapenem-resistant Enterobacterales, *Pseudomonas aeruginosa* and *A. baumannii* are increasingly represented and now constitute a central challenge for prevention and treatment (24, 25). These dynamic changes demand timely adaptation of empirical therapy, with early coverage of high-risk resistant Gram-negative organisms in appropriately selected patients, to mitigate treatment failure and poor outcomes.

Beyond microbiological characteristics, host-related factors play a crucial role in determining CRBSI prognosis (26). Disease severity scores such as Acute Physiology and Chronic Health Evaluation II (APACHE II), Sequential Organ Failure Assessment (SOFA) and Pitt bacteremia score are core components of prognostic assessment in severe infections, with higher scores consistently associated with increased short-term mortality (27–31). Specific MDROs, including MRSA, CR-KP and carbapenem-resistant *P. aeruginosa* (CRPA), have been identified as independent predictors of death in bloodstream infections (32, 33). Host-related parameters such as advanced age, hyperglycemia and hypoalbuminemia reflect baseline physiological reserve and nutritional status and have been incorporated as prognostic items in previous CRBSI-related scoring systems (34, 35). Dynamic changes in inflammatory biomarkers, including C-reactive protein (CRP) and procalcitonin (PCT), provide objective information on infection severity and treatment response (36, 37). In addition, the timeliness of key interventions—particularly catheter removal after onset of infection and the prompt initiation of targeted antimicrobial therapy according to susceptibility results—has been shown to significantly influence mortality, with delayed source control or optimization of antibiotics markedly increasing the risk of death (38, 39). Despite these insights, multivariable mortality prediction models that simultaneously incorporate disease severity, pathogen profile, host condition and intervention timing remain underdeveloped in the field of CRBSIs, and practical, user-friendly visual tools are still lacking (40).

Existing epidemiological data on CRBSIs also show marked geographical imbalance within China. Prior work has indicated that reports are disproportionately concentrated in northern, eastern and southwestern regions, whereas studies from southern China remain relatively scarce, partly due to uneven distribution of clinical research resources (41). In particular, regional data systematically linking different pathogen categories, including MDROs, to mortality risk in CRBSIs are limited.

Against this background, the present study focuses on patients with CRBSIs in a large tertiary hospital in Nanchang, southern China. We aimed to characterize the local epidemiology and pathogen distribution, to delineate the impact of different causative organisms—especially multidrug-resistant Gram-negative bacteria—on 28-day mortality, and to clarify the independent prognostic contributions of APACHE II score, cardiovascular disease (CVD) and other host-related factors. Furthermore, we sought to develop a visual, clinically applicable risk prediction model integrating multiple variables, with the goal of providing a regionally relevant tool to support early risk stratification, inform infection control strategies and guide individualized antimicrobial therapy in CRBSIs.

## Materials and methods

### Data collection

This study was a single-center retrospective cohort study conducted at the Second Affiliated Hospital of Nanchang University. Following approval by the institutional ethics committee, the research team retrieved and de-identified inpatient data from the hospital information system (HIS) and electronic health records (EHRs) between April 2024 and February 2025. The dataset included patients who were finally diagnosed with CRBSI from 1 January 2021, to 31 December 2024. All personally identifiable information was removed before data export, and the anonymized data were stored in a secured, encrypted database. After data verification and cleaning, statistical analyses were completed in June 2025.

The study protocol was approved by the Institutional Review Board of the Second Affiliated Hospital of Nanchang University (The study was reviewed and approved by the Medical Ethics Committee on 20 March 2024; no separate reference number was issued for expedited review projects). As the study involved only retrospective analysis of existing medical records and all patient identifiers were removed during data extraction, the ethics committee granted a waiver of written informed consent. The study was conducted in strict accordance with the Declaration of Helsinki and relevant data privacy regulations.

### Case definition

The definition of CRBSIs provided by the U.S. Centers for Disease Control and Prevention (CDC) includes the following criteria (42, 43), and patients were classified as having CRBSI only when they simultaneously fulfilled all of the following conditions: (i) hospitalization for more than 48 h; (ii) the presence of hypothermia (body temperature below 36.5 °C), hypotension (systolic blood pressure < 90 mmHg), fever, or chills; (iii) isolation of the same microorganism (with identical antimicrobial susceptibility patterns) from the central venous catheter (CVC), in the absence of any signs of local infection at the catheter insertion site, such as redness, irritation, or purulent discharge, with patients presenting such local signs classified as having exit-site or tunnel infection rather than CRBSI; time-to-positivity differences between catheter-drawn and peripheral blood cultures were not incorporated into the diagnostic criteria because these data were not consistently available in this retrospective cohort; (iv) no identifiable alternative source of infection. For patients with suspected CRBSI, CVC removal with catheter specimen culture was routinely performed when clinically feasible, and only those from whom a CVC specimen was obtained and who fulfilled criterion (iii) were included as confirmed CRBSI cases in this study. All suspected cases were independently reviewed by two infectious disease specialists, and a final diagnosis of CRBSI was established only when both evaluators agreed that all of the above criteria were fulfilled. Following confirmation of CRBSI diagnosis, patients were followed for 28 days by infectious disease specialists and designated hospital infection control personnel.

### Participant selection and study design

**FIGURE 1:**
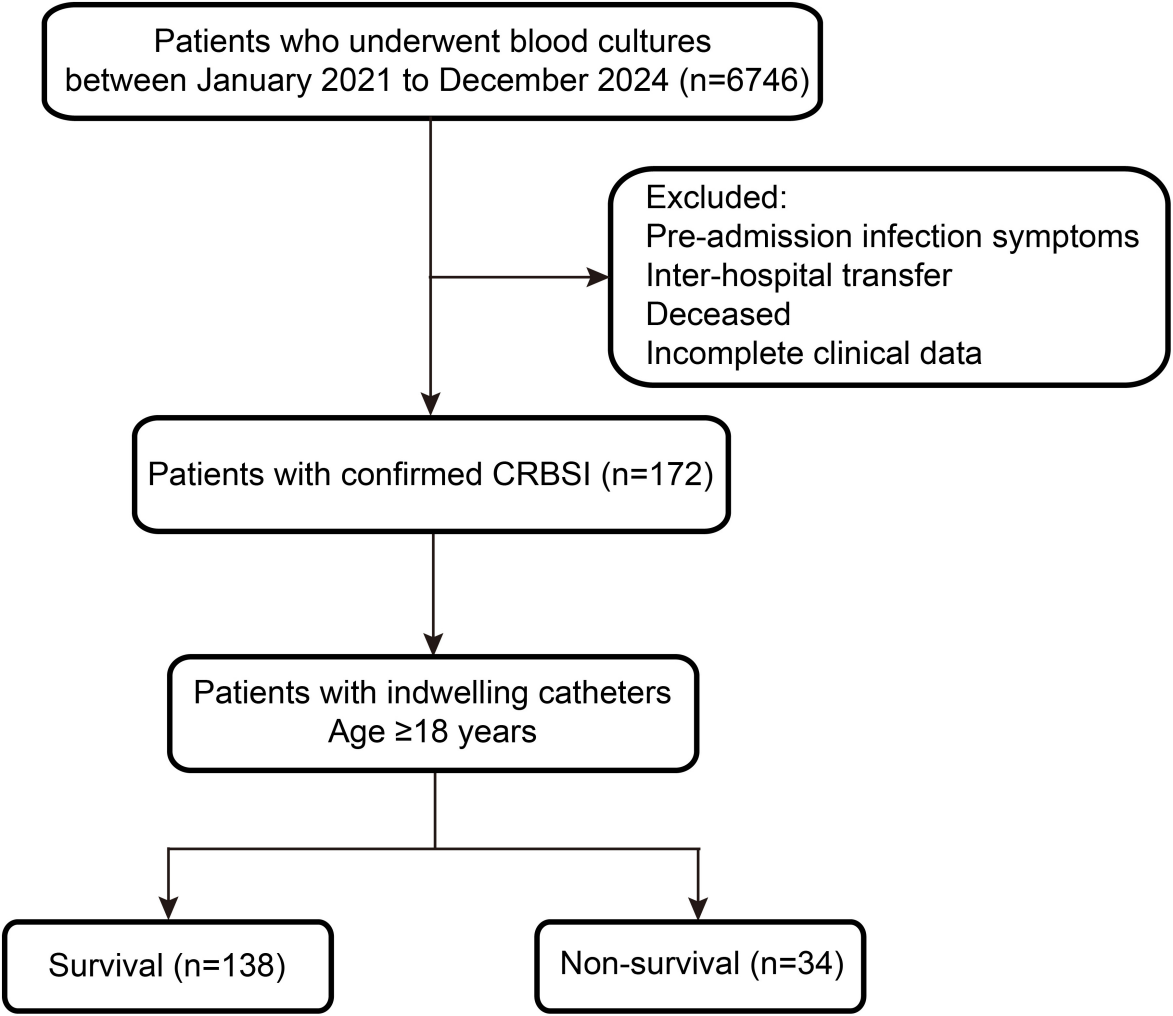
Flowchart of catheter-related bloodstream infection (CRBSI) screening and patient inclusion among hospitalized patients from 2021 to 2024.

The inclusion criteria for this study were as follows: (i) hospitalized patients diagnosed with CRBSIs during the study period at the research institution; (ii) patients aged ≥ 18 years; and (iii) patients with peripherally inserted central catheters (PICCs), CVCs, or hemodialysis catheters. The exclusion criteria were: (i) patients who presented with signs of infection (e.g., chills or fever) before admission; (ii) patients who were transferred to other hospitals, died, or discontinued treatment within 48 hours of admission; and (iii) patients with incomplete clinical data. Based on these inclusion and exclusion criteria, a total of 6,746 blood culture specimens were collected from 2021 to 2024. After excluding 6,574 non-CRBSI cases, 172 confirmed CRBSI cases were included in the final analysis, corresponding to an incidence rate of 0.27 per 1,000 catheter-days (Figure 1).

This retrospective cohort study employed sample size estimation using G*Power analysis based on preliminary data (44), with a statistical power of 80%. A total of 172 patients (including 34 deaths) were enrolled, meeting the required sample size. To investigate various risk factors influencing the prognosis of CRBSI patients, a 28-day follow-up was conducted. Based on clinical outcomes, patients were divided into two groups: survival and non-survival. Data analysis included multiple variables: age, gender, length of hospital stay, comorbidities (including diabetes, CVD, solid organ tumors, and cerebrovascular disease), hospital-related events (including department of admission at the time of infection, ICU admission within the past month, length of hospitalization before infection, and duration of catheter placement before CRBSI onset), and outcome measures (28-day mortality).

### Microbiological data

Based on patients’ clinical symptoms, both blood and catheter tip samples were collected for microbiological culture. Blood samples were processed using the fully automated BACT/ALERT^®^ 3D blood culture system (bioMérieux, Marcy-l’Étoile, France). Catheter tip specimens were collected using specialized sampling devices and transferred to culture plates for incubation. All isolated strains were identified using the VITEK^®^2 COMPACT system (bioMérieux), which employs a colorimetric method to detect microbial metabolic reactions to various substrates (45). Antimicrobial susceptibility testing (AST) was performed using the corresponding VITEK^®^2 AST cards. The minimum inhibitory concentration (MIC) was determined by broth microdilution, and results were interpreted according to the Clinical and Laboratory Standards Institute (CLSI) guidelines (2023 edition) (46). MDROs were monitored and included MRSA, carbapenem-resistant *P. aeruginosa* (CR-PA), CR-AB, CR-KP, vancomycin-resistant *Enterococcus* species, and carbapenem-resistant *Enterobacteriaceae*.

### Statistical analysis

All data were organized using Microsoft Excel and analyzed with SPSS version 26.0 (IBM Corp., USA). The normality of continuous variables was assessed using the Shapiro-Wilk test. Data with a normal distribution were expressed as mean ± standard deviation and compared between groups using independent sample *t*-tests. Non-normally distributed data were presented as medians (interquartile ranges, IQRs) and analyzed using the Mann-Whitney U test. Categorical variables were expressed as frequency (percentage), and group comparisons were performed using the chi-square test or Fisher’s exact test, as appropriate. Logistic regression analyses, including univariate and multivariate models, were conducted to identify associations with outcomes. A *p*-value < 0.05 was considered statistically significant.

## Results

### Patient baseline characteristics and epidemiology

A total of 172 cases of CRBSIs were identified between January 2021 and December 2024. The median patient age was 58.0 years (IQR: 48.25–70.75), and 95 patients (55.23%) were male. The median length of hospital stay before CRBSI onset was 12.5 days (IQR: 9–16), and the median duration of catheterization was 10 days (IQR: 6–16). The median APACHE II score was 9 (IQR: 7–12). Notably, 70 patients (40.70%) had been admitted to the ICU within one month before the CRBSI episode. CVD was the most common comorbidity, observed in 94 patients (54.65%), followed by diabetes (55 patients, 31.98%), solid tumors (40 patients, 23.26%), and respiratory diseases (36 patients, 20.93%) (Table 1). In laboratory testing, the median hemoglobin level was 78 g/L (IQR: 67.00–90.75), and the median serum albumin level was 30.94 g/L (IQR: 28.67–34.13). Regarding treatment history, 72 patients (41.86%) had received antibiotics within two weeks before CRBSI onset, and 85 patients (49.42%) had undergone surgery. Among these patients, prior antibiotics were most commonly prescribed for respiratory tract infections (including community-acquired and hospital-acquired pneumonia, as well as ventilator-associated pneumonia), followed by intra-abdominal or biliary infections and urinary tract infections. Of the 172 patients, 120 (69.8%) had their catheters inserted after admission and 52 (30.2%) already had a catheter in place at the time of admission, indicating that most catheters were placed after admission according to clinical needs rather than immediately on the first hospital day. The majority of catheters were CVC (54.07%) or hemodialysis catheters (41.86%). Microbiological testing revealed 89 cases (51.74%) of Gram-positive bacterial infections, 53 cases (30.81%) of Gram-negative bacterial infections, and 31 cases (18.02%) of fungal infections. The overall detection rate of MDROs was 19.19%, with CR-AB and MRSA each accounting for 7.56% of cases.

**TABLE 1:** Baseline characteristics of patients with CRBSIs regarding 28-day mortality.

Features	All (*n* = 172)	28-day mortality		*P*-value*
		Survival (*n* = 138)	Non-survival (*n* = 34)	
Age	58.0 (49.0–70.0)	58 (47.75–68.0)	57.0 (51.0–75.25)	0.087
Sex, male	95 (55.23)	79 (57.2)	16 (47.1)	0.285
Days of hospitalization before CRBSI	12.5 (9–16)	12.0 (9–16.0)	15.0 (9.75–18.25)	0.114
CL days before CRBSI	10 (6–16)	9 (6.75–15)	15 (5.75–18.25)	0.074
ICU admission 1 month prior	70 (40.70)	42 (30.4)	28 (82.4)	<*0.001*
Duration of hospitalization (days)	24 (19–33)	24 (19.25–32.75)	24 (19.75–37)	0.853
APACHE II score	9 (7–12)	8 (6–9)	19 (17–22)	<*0.001*
**Comorbidities**				
Cardiovascular diseases	94 (54.65)	68 (49.3)	26 (76.5)	*0.004*
Diabetes mellitus	55 (31.98)	37 (26.8)	18 (52.9)	*0.003*
Solid organ tumor	40 (23.26)	34 (24.6)	6 (17.6)	0.387
Respiratory system diseases	36 (20.93)	22 (15.9)	14 (41.2)	*0.001*
Coma	47 (27.33)	25 (18.1)	22 (64.7)	<*0.001*
**Laboratory findings**				
HB (g/L)	78 (67.00–90.75)	82.5 (70–94.25)	67.00 (56–72.75)	<*0.001*
ALB (g/L)	30.94 (28.67–34.13)	32.0 (29.0–35)	28.87 (27.0–30.69)	<*0.001*
**Treatment-related factors**				
Use of antibiotics within 2 weeks before CRBSI	72 (41.86)	42 (30.4)	30 (88.2)	<*0.001*
Surgical procedure	85 (49.42)	69 (50.0)	16 (47.1)	0.759
Catheter type				<*0.001*
CVC	93 (54.07)	62(44.9)	31 (91.2)	
PICC	7 (4.07)	7 (5.1)	0 (0)	
Hemodialysis tubes	72 (41.86)	69 (50.0)	3 (8.8)	
**Microorganisms**				
Gram-positive microorganisms	89 (51.74)	85 (61.6)	4 (11.8)	<*0.001*
Gram-negative microorganisms	53 (30.81)	37 (26.8)	16 (47.1)	*0.022*
Fungi	31 (18.02)	18 (13.0)	13 (38.2)	*0.002*
**MDROs**				
MRSA	13 (7.56)	13 (9.4)	0 (0)	0.074
CR-AB	13 (7.56)	7 (5.1)	6 (50.0)	<*0.001*
CR-KP	7 (4.07)	3 (2.2)	4 (17.6)	*0.002*

The symbol * indicates statistical significance, defined as *p* < 0.05. Data are displayed as *n* (%), medians (interquartile ranges), or means ± standard deviations. *P*-values represent the analytical comparison between 28-day survivors and non-survivors. *P*-values < 0.05 are shown in italics. CRBSI, catheter-related bloodstream infection; CL, central line; ICU, intensive care unit; APACHE, Acute Physiology and Chronic Health Evaluation; HB, hemoglobin level; ALB, albumin level; CVC, central venous catheter; PICC, peripherally inserted central catheters; MDRO, Multi-Drug Resistant Organisms; MRSA, methicillin-resistant *Staphylococcus aureus*; CR-AB, carbapenem-resistant *Acinetobacter baumannii*; CR-KP, carbapenem-resistant *Klebsiella pneumoniae*.

**FIGURE 2:**
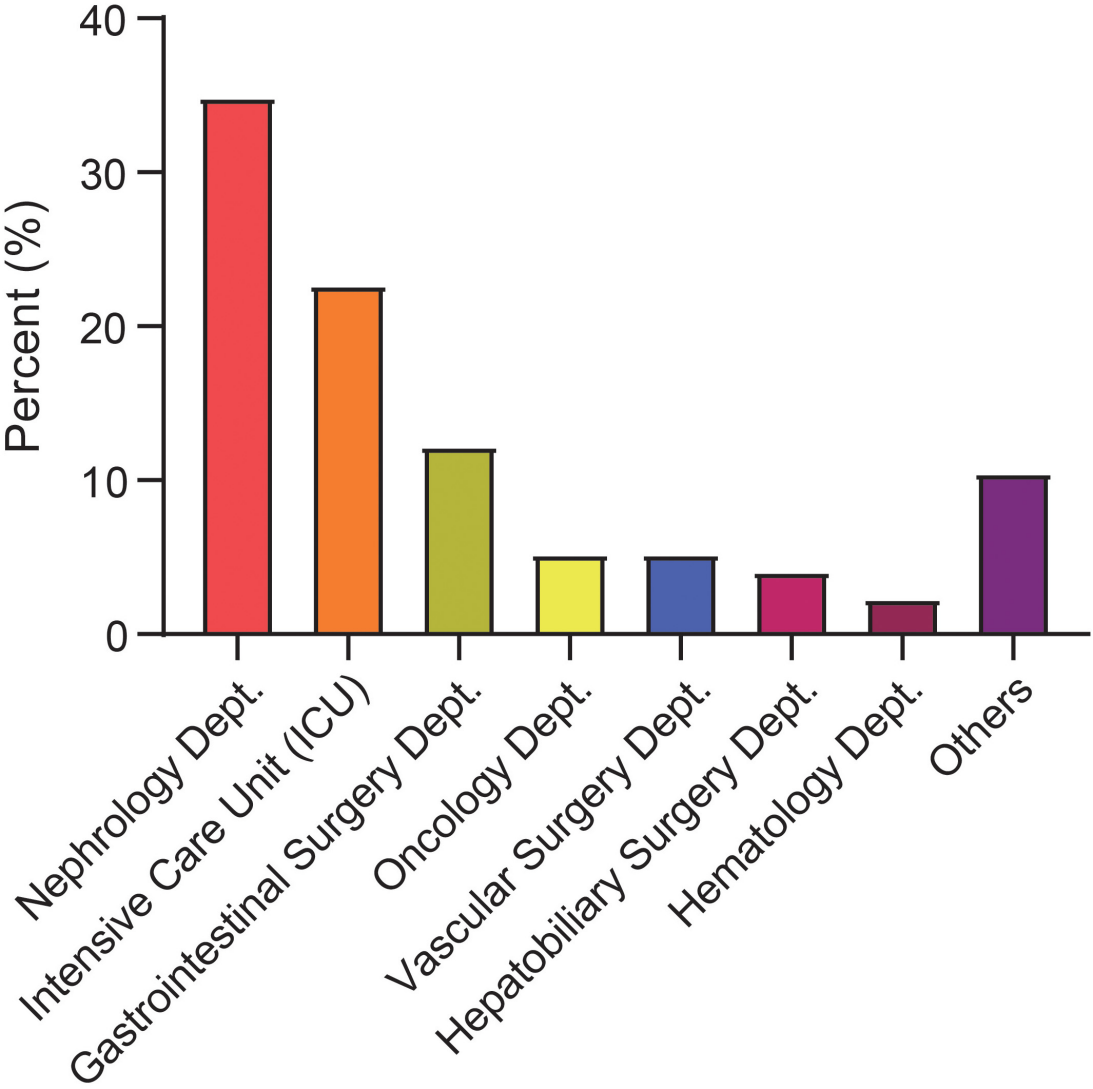
Distribution of catheter-related bloodstream infection (CRBSI) cases by clinical department from 2021 to 2024. Dept., department; ICU, intensive care unit.

In addition, this study systematically analyzed the epidemiological characteristics of CRBSIs from 2021 to 2024. The results demonstrated a significant year-by-year decline in CRBSI-related mortality (*p* = 0.024). Similarly, the overall incidence of CRBSIs, catheter-associated bloodstream infections, and hemodialysis CRBSIs showed a consistent downward trend during the study period (*p* < 0.01) (Supplementary Figure 1). This trend was closely associated with the implementation of strengthened CRBSI prevention strategies and policies, which promoted the rational use of antimicrobial agents at our institution. The distribution of CRBSIs varied across clinical departments. High-risk departments included the nephrology ward (34.88%), ICU (22.67%), and gastroenterology ward (12.21%) (Figure 2).

### Comparison of baseline characteristics between two groups of patients with CRBSIs

Between January 2021 and December 2024, a total of 172 cases of CRBSI were identified, resulting in an overall mortality rate of 19.77% (34/172). The analysis showed no significant differences between the survival group (*n* = 138) and the non-survival group (*n* = 34) in basic demographic characteristics such as gender and age (*p* > 0.05), or total hospital stay (both medians = 24 days, *p* = 0.853). However, clinical features indicated that the non-survival group had a more extended hospital stay (*p* = 0.114) and a longer catheter indwelling duration (*p* = 0.074) before CRBSI onset, although these differences did not reach statistical significance. Notably, 82.4% of patients in the non-survival group had been admitted to the ICU within one month before CRBSI diagnosis, compared to 30.4% in the survival group (*p* < 0.001). Severity of illness was significantly greater in the non-survival group, as reflected by higher APACHE II scores (*p* < 0.001). Regarding comorbidities, the non-survival group had significantly higher rates of CVD (*p* < 0.001), diabetes (*p* = 0.003), respiratory diseases (*p* = 0.001), and coma (*p* < 0.001). Laboratory data showed that hemoglobin and albumin levels were significantly lower in the non-survival group (both *p* < 0.001). Analysis of treatment-related factors revealed that 88.2% of patients in the non-survival group had received antibiotics within two weeks before CRBSI onset, compared to 30.4% in the survival group (*p* < 0.001). The use of CVC was also significantly more common in the non-survival group (91.2% vs. 44.9%, *p* < 0.001). Microbiological analysis indicated that Gram-positive bacterial infections were predominant in the survival group (61.6% vs. 11.8%, *p* < 0.001), while Gram-negative bacterial (47.1% vs. 26.8%, *p* = 0.022) and fungal infections (38.2% vs. 13.0%, *p* = 0.002) were significantly more frequent in the non-survival group. The detection rate of MDROs (e.g., CR-AB, CR-KP) was also markedly higher in the non-survival group (all *p* < 0.001) (Table 1).

### Microbiological analysis of CRBSI patients

**FIGURE 3:**
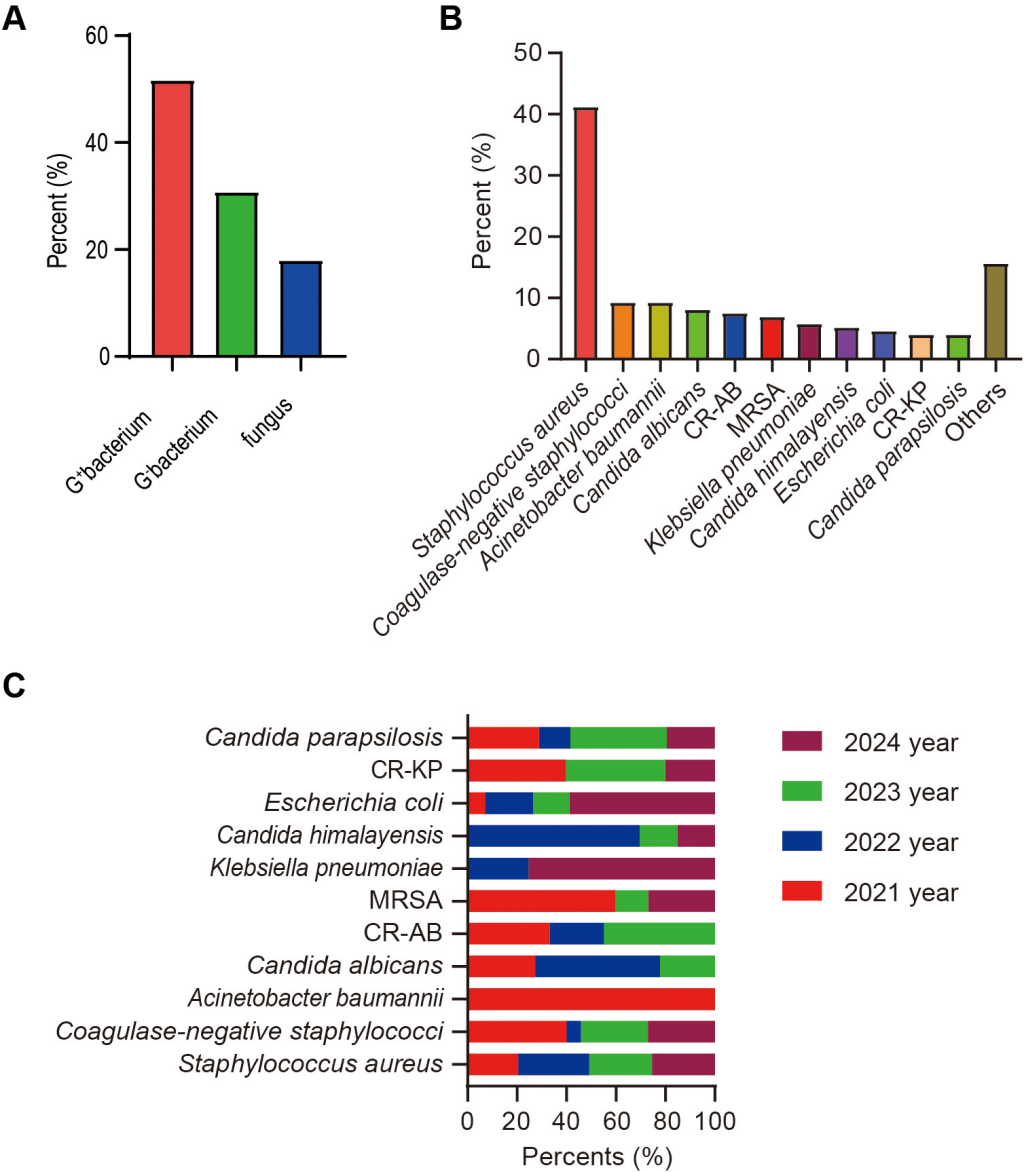
Distribution of catheter-related bloodstream infection (CRBSI) pathogens from 2021 to 2024. **(A)** Proportion of patients with bacterial and fungal infections. **(B)** Proportion of patients infected with each specific pathogen. **(C)** Temporal trends of pathogen distribution from 2021 to 2024. MRSA, methicillin-resistant *Staphylococcus aureus*; CR-AB, carbapenem-resistant *Acinetobacter baumannii*; CR-KP, carbapenem-resistant *Klebsiella pneumoniae*.

This study conducted a systematic analysis of the microbiological characteristics of 172 patients with CRBSIs. Due to a small number of polymicrobial infections, some cases could be classified into more than one pathogen category simultaneously. The results showed that Gram-positive bacteria were the most commonly isolated pathogens, accounting for 51.74% (89/172) of all isolates, followed by Gram-negative bacteria (30.81%, 53/172) and fungi (18.02%, 31/172) (Figure 3A). All Gram-positive isolates that met the CDC criteria for CRBSI in this cohort were Gram-positive cocci [predominantly *Staphylococcus aureus* and coagulase-negative staphylococci (CoNS)]; no Gram-positive bacilli were identified as causative pathogens, and all Gram-negative bacterial isolates were bacilli. *S. aureus* was identified as the predominant pathogen, present in 41.28% (71/172) of cases (Figure 3B). Notably, *Escherichia coli* and *K. pneumoniae* exhibited an increasing trend over the study period, whereas certain microorganisms, including *A. baumannii* and *Candida albicans*, showed a decline in prevalence (Figure 3C). Regarding antimicrobial resistance, MRSA accounted for 7.56% (13/172) of all isolates, with a decrease in its detection rate from 13.85% in 2021 to 6.25% in 2024. Among carbapenem-resistant pathogens, CR-AB (7.56%, 13/172) and CR-KP (4.07%, 7/172) were the predominant strains. The prevalence of CR-AB demonstrated a downward trend over time, potentially reflecting the effects of enhanced antimicrobial stewardship (Figures 3B, C).

### Analysis of factors associated with mortality in CRBSI patients

**FIGURE 4:**
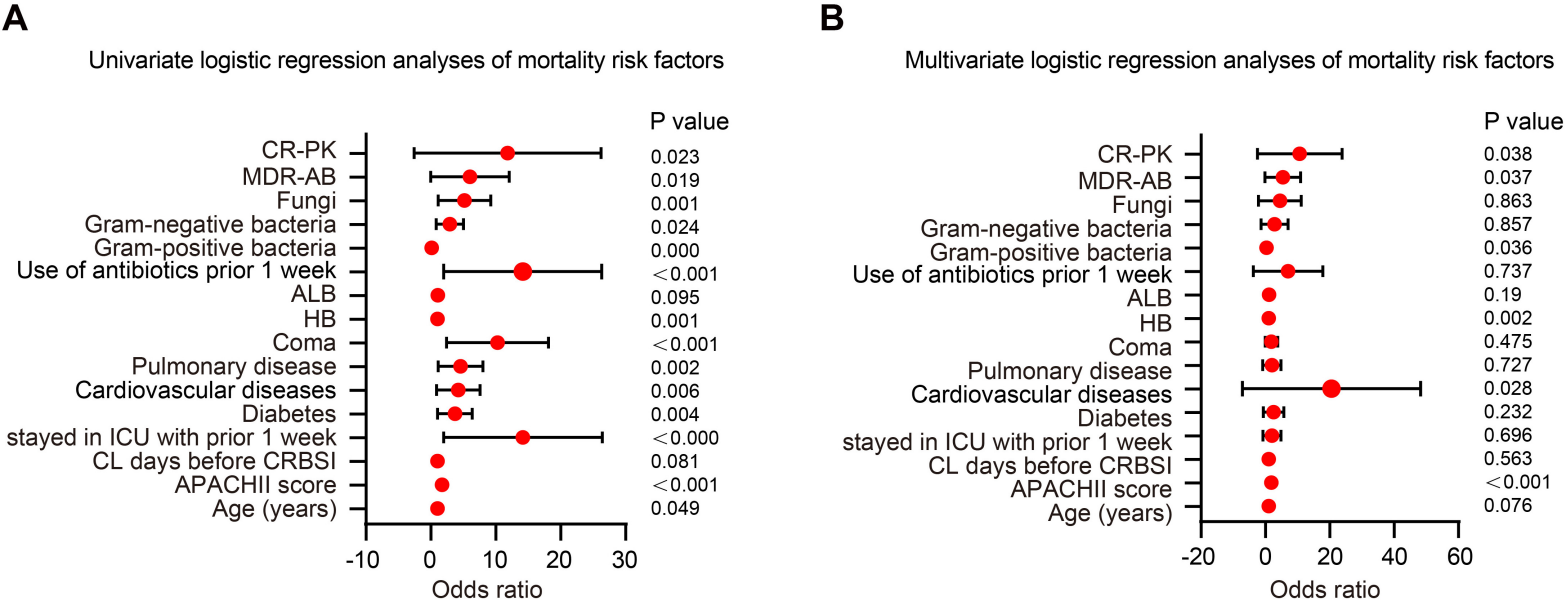
Logistic regression analysis of risk factors for mortality in catheter-related bloodstream infection (CRBSI) patients. **(A)** Univariate logistic regression analysis of mortality risk factors in CRBSI patients. **(B)** Multivariate logistic regression analysis of mortality risk factors in CRBSI patients.

Prognostic evaluation in patients with CRBSIs is critical for guiding clinical decision-making. In this study, logistic regression models were employed to systematically identify independent predictors of mortality in patients with CRBSI. Variables that showed statistically significant differences between the survival and non-survival groups were first analyzed using univariate logistic regression. Those with a significance level of *p* < 0.05 were then included in the multivariate logistic regression model for further analysis. The univariate logistic regression analysis revealed that several factors were significantly associated with mortality: age (OR = 1.027, 95% CI: 1.000–1.054, *p* = 0.049), APACHE II score (OR = 1.689, 95% CI: 1.435–1.988, *p* < 0.001), ICU admission (OR = 10.66, 95% CI: 4.101–27.774, *p* < 0.001), diabetes (OR = 3.071, 95% CI: 1.420–6.643, *p* = 0.004), CVD (OR = 3.346, 95% CI: 1.416–7.905, *p* = 0.006), pulmonary disease (OR = 3.691, 95% CI: 1.624–8.389, *p* = 0.002), coma (OR = 8.287, 95% CI: 3.628–18.929, *p* < 0.001), hemoglobin level (OR = 1.033, 95% CI: 1.013–1.054, *p* = 0.001), antibiotic use within two weeks before infection (OR = 10.667, 95% CI: 4.111–27.674, *p* < 0.001), Gram-negative bacterial infection (OR = 2.426, 95% CI: 1.122–5.249, *p* = 0.024), and fungal infection (OR = 4.127, 95% CI: 1.763–9.663, *p* = 0.001) (Table 2 and Figure 4A).

**TABLE 2:** Univariate and multivariate logistic regression analyses of risk factors for mortality in individuals who developed CRBSIs.

Variables	Univariate analysis		Multivariate analysis	
	OR (95% CI)	*P*-value	OR (95% CI)	*P*-value
Age (years)	1.027 (1.000–1.054)	0.049	1.093 (0.990–1.207)	0.076
APACHE II score	1.689 (1.435–1.988)	<0.001	1.771 (1.328–2.360)	<0.001
CLdays before CRBSI	1.031 (0.996–1.067)	0.081	1.029 (0.935–1.132)	0.563
ICU admission 1 month prior	10.66 (4.101–27.774)	<0.000	0.661 (0.226–5.274)	0.696
Diabetes mellitus	3.071 (1.420–6.643)	0.004	1.185 (0.226–6.21)	0.232
Cardiovascular diseases	3.346 (1.416–7.905)	0.006	8.076 (1.248–52.27)	0.028
Pulmonary disease	3.691 (1.624–8.389)	0.002	0.696 (0.091–5.313)	0.727
Coma	8.287 (3.628–18.929)	<0.001	1.735 (0.054–3.881)	0.475
HB	1.033 (1.013–1.054)	0.001	1.012 (1.016–1.047)	0.002
ALB	1.0503 (0.992–1.112)	0.095	1.131 (0.94–1.264)	0.19
Use of antibiotics 1 week prior	10.667 (4.111–27.674)	<0.001	1.543 (0.122–19.492)	0.737
**Microorganism**				
Gram-positive bacteria	0.084 (0.028–0.252)	0.000	0.081 (0.008–0.852)	0.036
Gram-negative bacteria	2.426 (1.122–5.249)	0.024	0.814(0.086–7.68)	0.857
Fungi	4.127 (1.763–9.663)	0.001	1.225(0.123–12.207)	0.863
**MDROs**				
CR-AB	4.010 (1.252–12.847)	0.019	3.549(1.081–11.653)	0.037
CR-KP	6.000 (1.276–28.222)	0.023	5.301(1.094–25.690)	0.038

The monitored strains of MDROs include *methicillin-resistant Staphylococcus aureus*, methicillin-resistant coagulase-negative Staphylococci, Vancomycin-resistant Enterococci, Carbapenem-resistant Enterobacteriaceae, Carbapenem-resistant *Pseudomonas aeruginosa*, Carbapenem-resistant *Acinetobacter baumannii*, and MDR-AB. CRBSI, catheter-related bloodstream infection; OR, odds ratio; CI, confidence interval; APACHE, Acute Physiology and Chronic Health Evaluation; CL, central line; ICU, intensive care unit; HB, hemoglobin level; ALB, albumin level; MDRO, Multi-Drug Resistant Organisms; MDR-AB, multi-drug resistant *Acinetobacter baumannii*; CR-KP, carbapenem-resistant *Klebsiella pneumoniae*.

In the multivariate analysis, the APACHE II score was significantly associated with an increased risk of mortality (OR = 1.771, 95% CI: 1.328–2.360; *p* < 0.001), indicating that disease severity was a key determinant of prognosis. Patients with cardiovascular comorbidities faced a markedly elevated risk of death (OR = 19.426, 95% CI: 1.248–52.270; *p* = 0.028), underscoring the prognostic importance of underlying health conditions. With respect to microbiological factors, infections caused by CR-AB (OR = 3.549, 95% CI: 1.081–11.653; *p* = 0.037) and CR-KP (OR = 5.301, 95% CI: 1.094–25.690; *p* = 0.038) were both associated with significantly increased mortality, highlighting the severe impact of MDRO infections. Notably, higher hemoglobin levels were also associated with increased mortality (OR = 1.012, 95% CI: 1.016–1.047; *p* = 0.002), a finding that may relate to hemoconcentration or transfusion-related complications and warrants further investigation. In contrast, Gram-positive bacterial infections demonstrated a protective effect (OR = 0.081, 95% CI: 0.008–0.852; *p* = 0.036), possibly due to greater susceptibility to standard antibiotic therapies (Table 2 and Figure 4B). Several variables that were statistically significant in univariate analysis (e.g., age, ICU admission, and diabetes) were no longer significant after multivariable adjustment, indicating that their apparent associations may be attenuated by confounding, particularly disease severity. In contrast, among microbiological/MDRO-related variables, CR-AB and CR-KP remained independently associated with 28-day mortality, whereas other monitored MDROs (e.g., MRSA) were uncommon and had no/very few events in the non-survival group, limiting statistical power and precluding reliable effect estimation in multivariable models. Collectively, these findings support risk stratification and individualized management for patients with CRBSIs.

### Mortality risk prediction and clinical value in CRBSI patients

**FIGURE 5:**
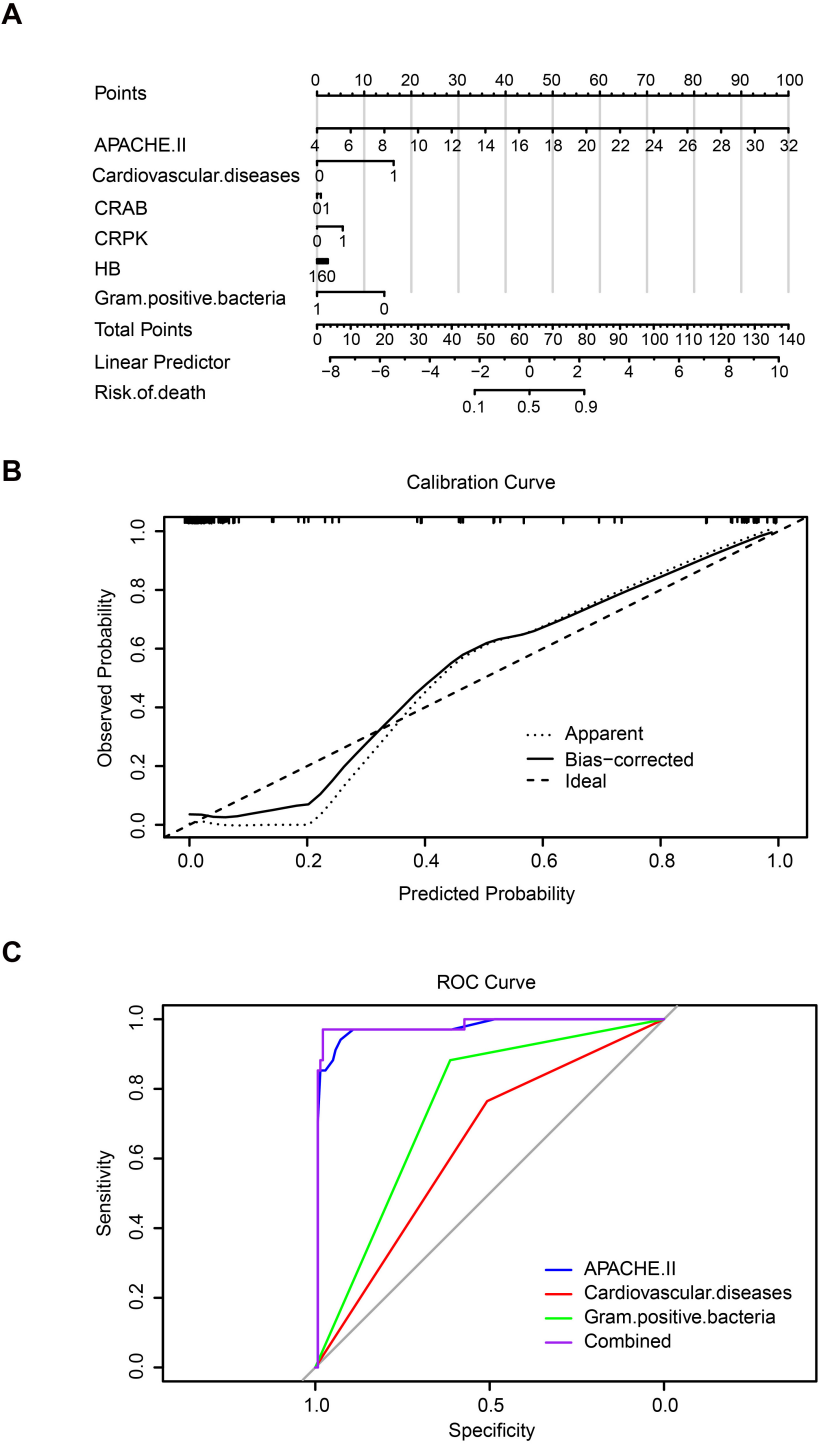
Predictive assessment of mortality risk in catheter-related bloodstream infection (CRBSI) patients. **(A)** Nomogram model for quantifying mortality risk in CRBSI patients. **(B)** Calibration curve of the model. **(C)** Receiver operating characteristic (ROC) curve analysis.

Furthermore, a nomogram model was constructed based on multivariate logistic regression to quantitatively assess the risk of death in patients with CRBSIs. The results indicated that the APACHE II score was the most influential predictor, exhibiting the widest point scale; higher APACHE II values were associated with an increased risk of mortality in patients with CRBSI. Cardiovascular complications and Gram-positive bacterial infections also contributed significantly to the model. Specifically, cardiovascular complications increased the risk of mortality, whereas Gram-positive bacterial infections were associated with a decreased risk. Other variables, including CR-AB, CR-KP infections, and hemoglobin levels, had relatively shorter point axes, suggesting a smaller contribution to overall risk prediction (Figure 5A). The nomogram demonstrated excellent predictive performance, with a concordance index (C-index) of 0.979. The calibration curve demonstrated good agreement between the predicted probabilities and the observed outcomes, indicating a strong model fit (Figure 5B). Receiver operating characteristic (ROC) curve analysis further confirmed the model’s strong discriminatory capacity, with an area under the curve (AUC) of 0.979. Notably, the predictive ability of the APACHE II score alone closely approached that of the combined model (AUC = 0.973), reaffirming that disease severity was the central determinant of mortality risk. Gram-positive bacterial infections, as a protective factor, exhibited moderate predictive value (AUC = 0.748), whereas CVD demonstrated relatively limited independent predictive power (AUC = 0.636). At the optimal cutoff point, the model achieved a sensitivity of 92.3% and a specificity of 91.8%, indicating excellent clinical discrimination (Figure 5C).

## Discussion

This study, conducted in a large tertiary referral hospital in Nanchang, analyzed 172 patients with CRBSIs, systematically characterizing their epidemiological features, microbiological profiles and independent predictors of 28-day mortality, thereby providing local evidence to support regional CRBSI management and prognostic assessment. We observed a year-on-year decline in CRBSI incidence from 2021 to 2024, a trend likely linked to the implementation of catheter care bundles, reinforced hand hygiene and antimicrobial stewardship at national and institutional levels (47). Despite the overall decline, CRBSIs remained clustered in nephrology, the ICU and gastroenterology. This pattern is consistent with previous work identifying the ICU as a primary site of CRBSI (48), while also underscoring that other catheter-intensive wards should not be overlooked. In nephrology, catheter utilization is extremely high among hemodialysis patients, with more than 85% initiating dialysis via central venous catheters and a reported CRBSI incidence of approximately 0.78/1,000 catheters (49). In the ICU, central venous catheters are almost routine for life support and hemodynamic monitoring, with a CRBSI incidence of about 9.63% (50). Patients in gastroenterology and gastrointestinal surgery similarly require long-term vascular access for chemotherapy or perioperative support, with reported CRBSI incidences of 10.82%–12.94% and 8.0%, respectively (51, 52). Together, these data extend the traditional ICU-centric view of CRBSI risk and highlight the need to prioritize strengthened catheter management strategies across all high-utilization departments.

With respect to microbiology, Gram-positive bacteria remained the predominant pathogens, accounting for more than half of all isolates, with *S. aureus* as the most common species. This finding is in line with most domestic studies and reinforces the central role of Gram-positive organisms in bloodstream infections (53, 54). Compared with classic CRBSI series in which CoNS predominate (55), our cohort showed a higher proportion of *S. aureus* and a lower proportion of CoNS. This discrepancy is plausibly explained by the strict CDC-based diagnostic criteria applied in this study, which likely excluded a larger proportion of CoNS-related blood culture contaminants and therefore yielded a pathogen spectrum more reflective of true infection. In addition, the high proportion of surgical and hemodialysis patients in our cohort, with frequent disruption of skin barriers, may favor bloodstream invasion by high-virulence *S. aureus*. Notably, we also observed a progressive increase in Gram-negative pathogens—particularly *E. coli* and *K. pneumoniae*—over the study period, a trend consistent with longitudinal data from large urban hospitals (56, 57). Intensive use of broad-spectrum agents, especially third-generation cephalosporins, can deplete susceptible intestinal flora and select for resistant Enterobacterales (e.g., ESBL-producing strains), promoting their colonization (58). In the context of increasing invasive procedures and a growing population of critically ill patients, more frequent disruption of physiological barriers facilitates endogenous translocation of these colonizing resistant Gram-negative bacilli into the bloodstream, thereby increasing their contribution to the CRBSI pathogen spectrum (12). In this setting, effective infection control requires parallel surveillance of both Gram-positive and Gram-negative trends to strengthen early warning systems and refine empiric antimicrobial strategies.

In this cohort, the 28-day all-cause mortality of CRBSIs was 19.77%, slightly higher than some domestic reports but still acceptable for a high-risk tertiary population. Further analysis demonstrated that the APACHE II score was the strongest independent predictor of mortality, underscoring the central role of baseline disease severity, consistent with its established prognostic value in critically ill patients (27–29). Unlike previous studies that typically evaluated APACHE II in isolation, we incorporated APACHE II, cardiovascular comorbidity and microbiological variables into a nomogram, and confirmed its predictive performance by ROC analysis (C-index = 0.979). This approach not only reaffirms the clinical utility of APACHE II, but also provides a more intuitive and practical tool for bedside risk stratification in specific infectious populations.

Beyond global severity, we identified CVD as another independent risk factor for 28-day mortality, addressing a gap in prior CRBSI prognostic models that have rarely included CVD (59). Although chronic comorbidities have been broadly associated with adverse infection outcomes (60, 61), our findings emphasize the particular impact of CVD in the context of CRBSIs: infection-related systemic inflammation and hemodynamic instability can exacerbate pre-existing cardiac dysfunction, precipitate multi-organ injury and ultimately increase mortality. This suggests that, in patients with CRBSIs and CVD, management should shift from a narrow focus on source control and antimicrobial therapy toward an integrated strategy that balances “infection control and cardiovascular protection,” with meticulous hemodynamic management, cardioprotective care and early cardiology involvement, especially in elderly patients with limited cardiac reserve.

A clinically informative observation from our multivariable analysis was that Gram-positive infection, considered as a composite category, was inversely associated with mortality and appeared as a “protective” factor. This does not imply that Gram-positive bacteremia is benign, nor that it is associated with shorter time-to-positivity or easier localization of the infectious focus. Rather, the association likely reflects differences in therapeutic accessibility and pathogen biology. Many common Gram-positive pathogens, such as methicillin-susceptible *S. aureus* (MSSA) and some CoNS, remain susceptible to β-lactams and glycopeptides, which are widely available and well-characterized pharmacodynamically, enabling more predictable treatment responses (62, 63). In addition, several Gram-positive species (e.g., *S. epidermidis*) are intrinsically less virulent, whereas Gram-negative bacilli release endotoxin that can trigger more intense systemic inflammation and septic shock, leading to more severe organ damage (64–66). Our results are therefore consistent with reports suggesting that, when recognized early and appropriately covered empirically, Gram-positive infections may be relatively more manageable (67). At the same time, substantial heterogeneity exists within the Gram-positive group: *S. aureus* (including MRSA and MSSA) produces potent virulence factors such as coagulase and often causes highly invasive disease (68), and MRSA’s broad β-lactam resistance increases therapeutic complexity and has been associated with worse outcomes than MSSA (69, 70). CoNS, although generally less virulent, possess strong biofilm-forming capacity and are prototypical pathogens of device-related infections (71). Owing to limited event numbers and sample size, we were unable to perform adequately powered stratified analyses comparing MRSA, MSSA and individual CoNS species; this important question warrants further investigation in larger cohorts.

The decline in MRSA detection observed in our study is likely driven by multiple concurrent interventions. Strengthened infection control measures, such as improved hand hygiene, contact precautions and environmental cleaning, can reduce nosocomial MRSA transmission, while antimicrobial stewardship programmes mitigate selection pressure by limiting unnecessary anti-staphylococcal antibiotic use. In addition, MRSA screening and decolonization strategies implemented in some institutions have been shown to reduce MRSA infection burden (72–74). The convergence of these measures may underlie the downward trend in MRSA-related CRBSIs at our center and illustrates how coordinated infection prevention and antimicrobial stewardship can reshape pathogen ecology over time.

In contrast to the relatively favorable outcomes associated with Gram-positive infections overall, CR-KP and CR-AB were clearly identified as independent risk factors for 28-day mortality, reflecting the high lethality of these MDROs. CR-KP and CR-AB frequently harbor multiple resistance mechanisms, dramatically narrowing the spectrum of effective agents, and often exhibit strong biofilm-forming capacity, facilitating persistent colonization of catheters and hospital environments and leading to protracted, difficult-to-eradicate infections (75, 76). The rising trend of CR-KP in Nanchang parallels that reported in coastal cities such as Guangzhou and Fuzhou (77), suggesting a risk of regional dissemination of resistance. In this context, our findings support a pathogen-specific management strategy: for CRBSIs caused by CR-KP or CR-AB, prompt catheter removal and thorough source control should be prioritized; individualized combination antibiotic regimens should be initiated as early as possible based on susceptibility testing; and strict contact precautions should be implemented to prevent nosocomial spread of MDROs (77–79). More broadly, our data indicate that clinicians should pay particular attention to patients with high APACHE II scores, concomitant CVD and CR-GNB infection, especially in nephrology and ICU settings where catheter density and MDRO pressure are highest.

This study has several limitations. First, this was a single-center retrospective cohort conducted in a tertiary referral hospital with a high proportion of critically ill and transferred patients; therefore, selection bias is unavoidable and the generalizability of our findings may be greatest in similar high-risk settings rather than in community hospitals or general wards. Second, although key catheter-related variables were available, more granular information (e.g., insertion site, number of lumens, catheter care bundle adherence, timing of catheter removal/replacement, and other procedural details) was not consistently recorded in the electronic charts. In addition, several time-sensitive diagnostic and therapeutic parameters, such as differential time to positivity between catheter-drawn and peripheral blood cultures, semi-quantitative catheter-tip cultures, and the timing/appropriateness of initial empirical antimicrobial therapy, were incompletely documented, which may have introduced residual confounding and potential misclassification, particularly for CoNS-associated episodes. Third, the non-survival group was relatively small (34 deaths), which limited the statistical power for exploring rare pathogens and finer subgroups (e.g., MRSA vs. MSSA vs. CoNS) and may have affected the stability of regression estimates. Finally, although the nomogram showed high discrimination (C-index = 0.979), it was only internally validated within the same cohort and may be subject to optimism and overfitting; external validation and calibration in larger multicentre prospective cohorts are required before broad clinical implementation.

## Conclusion

This study systematically characterized the clinical features and prognostic determinants of CRBSIs, providing critical evidence for risk stratification and therapeutic decision-making. We identified the APACHE II score, underlying CVD and infection with CR-KP or CR-AB as independent predictors of 28-day mortality, and showed that, at the grouped level, Gram-positive infections were associated with comparatively more favorable outcomes. These findings translate into several concrete clinical implications: clinicians should routinely calculate APACHE II at CRBSI onset and use the proposed nomogram to identify high-risk patients early; patients with concomitant CVD warrant intensified hemodynamic monitoring and cardiology co-management; episodes caused by CR-KP or CR-AB should prompt immediate catheter removal, early initiation of active (often combination) antimicrobial regimens and strict contact precautions; and catheter care bundles together with targeted surveillance for carbapenem-resistant Gram-negative organisms should be prioritized in high-utilization wards such as nephrology and the ICU. Implementation of these measures may help to optimize antimicrobial use, focus resources on patients at greatest risk and ultimately improve the short-term prognosis of CRBSI.

## Data Availability

The raw data supporting the conclusions of this article will be made available by the authors, without undue reservation.

## Supplementary material

Supplementary Figure 1 Incidence rates of CRBSIs per 1,000 catheter-days. **(A)** CRBSI incidence per 1,000 catheter-days among hospitalized patients from 2021 to 2024. **(B)** Incidence of CVC-related bloodstream infections per 1,000 catheter-days from 2021 to 2024. **(C)** Incidence of hemodialysis CRBSIs per 1,000 catheter-days from 2021 to 2024. **(D)** Incidence of PICC-related bloodstream infections per 1,000 catheter-days from 2021 to 2024. CRBSI: catheter-related bloodstream infection; CVC, central venous catheter; PICC, peripherally inserted central catheter.
