# Supplementary figures and images for: Dynamic shifts in pathogen ecology of catheter-related bloodstream infections: temporal trends and ward-specific risk landscapes
Source: Front Med (Lausanne). 2026 Jan 23;12:1665350. doi: 10.3389/fmed.2025.1665350 (PMC12879051; doi:10.3389/fmed.2025.1665350)

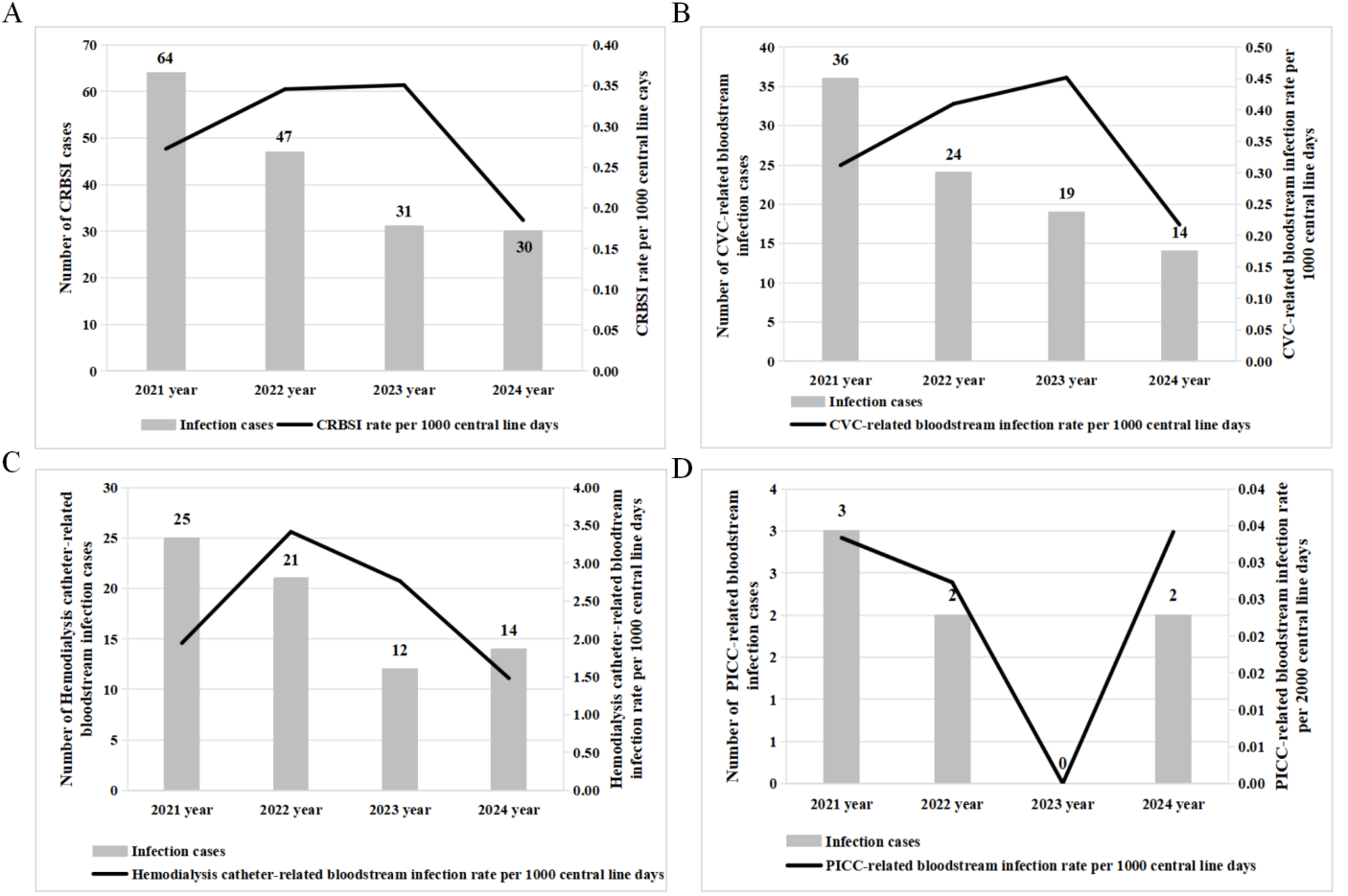

Supplement: Supplementary Figure 1 — Incidence rates of CRBSIs per 1,000 catheter-days. (A) CRBSI incidence per 1,000 catheter-days among hospitalized patients from 2021 to 2024. (B) Incidence of CVC-related bloodstream infections per 1,000 catheter-days from 2021 to 2024. (C) Incidence of hemodialysis CRBSIs per 1,000 catheter-days from 2021 to 2024. (D) Incidence of PICC-related bloodstream infections per 1,000 catheter-days from 2021 to 2024. CRBSI: catheter-related bloodstream infection; CVC, central venous catheter; PICC, peripherally inserted central catheter. [file Image_1.png]
